# A Novel Small Molecule Neurotrophin-3 Analogue Promotes Inner Ear Neurite Outgrowth and Synaptogenesis *In vitro*

**DOI:** 10.3389/fncel.2021.666706

**Published:** 2021-07-15

**Authors:** Judith S. Kempfle, Marlon V. Duro, Andrea Zhang, Carolina D. Amador, Richard Kuang, Ryan Lu, Boris A. Kashemirov, Albert S. Edge, Charles E. McKenna, David H. Jung

**Affiliations:** ^1^Department of Otolaryngology, Massachusetts Eye and Ear Infirmary, Harvard Medical School, Boston, MA, United States; ^2^Department of Otolaryngology, University Medical Center Tübingen, Tübingen, Germany; ^3^Department of Chemistry, University of Southern California, Los Angeles, CA, United States

**Keywords:** inner ear, regeneration, synaptopathy, neurotrophin-3, bisphosphonate, small molecule, hidden hearing loss, sensorineural hearing loss

## Abstract

Sensorineural hearing loss is irreversible and is associated with the loss of spiral ganglion neurons (SGNs) and sensory hair cells within the inner ear. Improving spiral ganglion neuron (SGN) survival, neurite outgrowth, and synaptogenesis could lead to significant gains for hearing-impaired patients. There has therefore been intense interest in the use of neurotrophic factors in the inner ear to promote both survival of SGNs and re-wiring of sensory hair cells by surviving SGNs. Neurotrophin-3 (NT-3) and brain-derived neurotrophic factor (BDNF) represent the primary neurotrophins in the inner ear during development and throughout adulthood, and have demonstrated potential for SGN survival and neurite outgrowth. We have pioneered a hybrid molecule approach to maximize SGN stimulation *in vivo*, in which small molecule analogues of neurotrophins are linked to bisphosphonates, which in turn bind to cochlear bone. We have previously shown that a small molecule BDNF analogue coupled to risedronate binds to bone matrix and promotes SGN neurite outgrowth and synaptogenesis *in vitro*. Because NT-3 has been shown in a variety of contexts to have a greater regenerative capacity in the cochlea than BDNF, we sought to develop a similar approach for NT-3. 1Aa is a small molecule analogue of NT-3 that has been shown to activate cells through TrkC, the NT-3 receptor, although its activity on SGNs has not previously been described. Herein we describe the design and synthesis of 1Aa and a covalent conjugate of 1Aa with risedronate, Ris-1Aa. We demonstrate that both 1Aa and Ris-1Aa stimulate neurite outgrowth in SGN cultures at a significantly higher level compared to controls. Ris-1Aa maintained its neurotrophic activity when bound to hydroxyapatite, the primary mineral component of bone. Both 1Aa and Ris-1Aa promote significant synaptic regeneration in cochlear explant cultures, and both 1Aa and Ris-1Aa appear to act at least partly through TrkC. Our results provide the first evidence that a small molecule analogue of NT-3 can stimulate SGNs and promote regeneration of synapses between SGNs and inner hair cells. Our findings support the promise of hydroxyapatite-targeting bisphosphonate conjugation as a novel strategy to deliver neurotrophic agents to SGNs encased within cochlear bone.

## Introduction

Sensorineural hearing loss (SNHL), is associated with loss of cochlear outer hair cells (OHCs), inner hair cells (IHCs), and spiral ganglion neurons (SGNs; Schuknecht and Gacek, 1993; Wu et al., 2019, 2020). The afferent ribbon synapse between IHCs and SGNs appears to be the element most sensitive to noise in rodents and non-human primates (Kujawa and Liberman, 2006, 2009; Valero et al., 2017). In rodents, such loss is associated with an over-compensation in central gain (Chambers et al., 2016). Although the clinical consequences of ribbon synapse loss in human patients remain under investigation (Bramhall et al., 2014), it is therefore reasonable to suspect that such loss could be linked with other symptoms, including tinnitus, hyperacusis, and difficulty hearing in background noise (Auerbach et al., 2014; Bharadwaj et al., 2015; Chambers et al., 2016; Liberman and Kujawa, 2017).

After a synaptic loss, the cell bodies of IHCs and SGNs remain present for months in mice and, potentially, for decades in humans (Sergeyenko et al., 2013; Viana et al., 2015; Wu et al., 2019). The prolonged survival of these cell bodies suggests a therapeutic window for exogenous neurotrophic treatment to promote synapse regeneration. Two major inner ear neurotrophins, neurotrophin-3 (NT-3) and brain-derived neurotrophic factor (BDNF) are essential for wiring during SGN development and for SGN survival postnatally (Ernfors et al., 1995; Bianchi et al., 1996; Silos-Santiago et al., 1997; Stankovic et al., 2004; Green et al., 2012). Postnatally, BDNF and NT-3 are mainly expressed in supporting cells and hair cells and interact with their respective tropomyosin receptor kinase receptors (Trks), TrkB for BDNF, and TrkC for NT-3, which are expressed by SGNs. While BDNF and NT-3 are expressed in the adult cochlea (Bailey and Green, 2014), their endogenous levels do not appear to be sufficient to induce synaptic regeneration following noise damage (Kujawa and Liberman, 2006; Valero et al., 2017) or to preserve synapses during aging (Sergeyenko et al., 2013).

An intense investigation has therefore focused on delivering exogenous neurotrophic activities to the inner ear as a treatment for hearing loss due to ribbon synapse or SGN deficiency. Experiments using genetically modified mice suggest that overexpression of NT-3, but not BDNF, improves cochlear responses and regenerates ribbon synapses after noise damage (Wan et al., 2014). As with any inner ear therapy, however, neurotrophin entry into the labyrinth presents a barrier. A variety of methods have therefore been utilized to directly introduce neurotrophin protein into the cochlea (Noushi et al., 2005; Evans et al., 2009). Expression of neurotrophin protein within the cochlea *via* a viral vector, which similarly requires a labyrinthotomy, has also been reported (Ramekers et al., 2015; Chen et al., 2018; Akil et al., 2019; Hashimoto et al., 2019). However, intracochlear viral overexpression of neurotrophin has also been found to be ototoxic, potentially limiting this approach (Fukui and Raphael, 2013; Akil et al., 2019; Hashimoto et al., 2019). With respect to a non-cochleoinvasive approach, direct application of NT-3 protein on the round window membrane has been reported, with variable results (Sly et al., 2016; Suzuki et al., 2016). In this regard, transport across the RWM is highly variable, and transit of proteins in particular across the RWM is thought to be inefficient (Goycoolea, 2001).

Small molecules that mimic neurotrophin function and act on Trk receptors have therefore been recognized for their potential in preventing neural cell death and supporting neural survival (Lewis et al., 2006; Jang et al., 2007; Lin et al., 2007; Price et al., 2007). With respect to local cochlear delivery, such compounds could offer a promising alternative by promoting diffusion across the RWM, resulting in higher concentration within the perilymph and eliminating the hearing and balance risk associated with a labyrinthotomy (Hao and Li, 2019; Nyberg et al., 2019). Agonists of TrkB and TrkC have been identified, including 7,8-dihydroxyflavone (DHF) for TrkB (Bai et al., 2010; Jang et al., 2010), and 1Aa for TrkC (Zaccaro et al., 2005; Peleshok and Saragovi, 2006). The neurotrophic effect of DHF has been extensively studied in other contexts. DHF treatment protects against neural degeneration in various disease models and promotes neurogenesis in the hippocampus (He et al., 2016; Garcia-Diaz Barriga et al., 2017; Stagni et al., 2017; Aytan et al., 2018). With respect to SGNs, we and others have demonstrated that DHF has neurotrophic effects on SGNs leading to improved neuron survival, axon outgrowth, and synaptic regeneration *in vitro* (Jang et al., 2010; Kramer et al., 2017; Kempfle et al., 2018) and *in vivo* (Fernandez et al., 2021). 1Aa was first identified in a screen of small molecule TrkC agonist peptidomimetics (Zaccaro et al., 2005). However, the neurotrophic activity of a small molecule NT-3 analogue on SGNs has not previously been described.

To promote cochlear drug delivery, we have developed a platform aimed at the exploitation of cochlear bone as a depot for prolonged neurotrophic stimulation of SGNs. In this regard, we have previously demonstrated that a fluorescently-labeled bisphosphonate can cross the RWM in a non-ototoxic manner and bind cochlear bone (Kang et al., 2015). We then described the synthesis of a hybrid small molecule, Ris-DHF, that linked DHF to risedronate (Ris), a bisphosphonate with high bone mineral affinity. Ris-DHF demonstrated strong neurotrophic activity on SGNs, both when free and when bound to hydroxyapatite bone matrix (Kempfle et al., 2018). Here, we utilize similar synthetic chemistry to conjugate a bisphosphonate to a small molecule NT-3 analogue. We synthesized the small molecule TrkC agonist 1Aa (Pattarawarapan et al., 2002; Lee et al., 2004; Zaccaro et al., 2005) and a conjugate of Ris and 1Aa (Ris-1Aa) to study their ability to promote neurite outgrowth and ribbon synapse regeneration *in vitro*.

## Materials and Methods

### General Chemistry

Dry dichloromethane (DCM) was prepared by distillation over CaH_2_. Anh. methanol (MeOH) and *N,N*-dimethylformamide (DMF) were purchased from Merck. 2,6-Lutidine and *N*,*N*-diisopropylethylamine (DIEA) were distilled over KOH. 2-Fluoro-5-nitrobenzoyl chloride (Jackman et al., 1990), 4-({[(9*H*-fluoren-9-yl)methoxy]carbonylamino})butanoic acid (Fmoc-GABA-OH; Aronov et al., 2004), and 1-(3-amino-2-hydroxypropyl)-3-(2-hydroxy-2, 2-diphosphonoethyl)pyridin-1-ium (RIS-linker; Kempfle et al., 2018) were prepared following literature procedures. All other reagents and solvents were obtained from commercial sources and used without further purification. Reactions on solid support were performed in a fritted polypropylene syringe (5 ml, Torviq) at room temperature using a manual control shaking apparatus (VWR OS-500) and standard Fmoc chemistry on Rink amide resin [(4-(2′,4′-dimethoxyphenyl-Fmoc-aminomethyl)phenoxy resin, Advanced ChemTech, SA5030, 100–200 mesh, 0.5 mmol/g)]. Removal of the Fmoc group was monitored by UV spectroscopy (288 and 299 nm) and capping of primary NH_2_ groups was monitored by the Kaiser test (ninhydrin). General purification and characterization procedures were done as described previously (Kempfle et al., 2018).

#### Preparation of Template Compound

##### 4-(Azidomethyl)-2-nitrobenzoic Acid (1)

To a solution of 4-(bromomethyl)-3-nitrobenzoic acid (502 mg, 1.93 mmol) in anh. DMF (9.6 ml) was slowly added sodium azide (1 equiv, 125 mg) and reacted at room temperature overnight, under nitrogen atmosphere. The crude reaction mixture was then extracted with ethyl acetate (2 × 20 ml) and 0.1 M aq. HCl (2 × 20 ml). The organic solvent was dried over anh. MgSO_4_ and evaporated under reduced pressure. The desired azide **1**, an orange oil, was obtained, with a quantitative yield, and used immediately in the next step without further purification (Supplementary Figures 2–5). ^1^H NMR (400 MHz, methanol-*d*_4_) δ 8.55 (s, ^1^H), 8.25 (d, *J* = 8.1 Hz, ^1^H), 7.78 (d, *J* = 8.0 Hz, ^1^H), 4.89 (s, 2H). ^13^C NMR (101 MHz, Methanol-*d*_4_) δ 165.40, 147.69, 135.86, 134.05, 131.83, 130.43, 125.59, 51.36. MS (ESI) *m/z*: [M–H]^−^ calcd for C_8_H_5_N_4_O_4_^−^ 221.0; found: 221.2.

##### 4-(Aminomethyl)-3-nitrobenzoic Acid (2)

The azide **1** (1.93 mmol) was dissolved in 1.6 ml of water and 5.1 ml of THF and triphenylphosphine (1.4 equiv, 709 mg) was slowly added to the reaction mixture and reacted at room temperature overnight (Marinzi et al., 2004; Xu et al., 2011). The solvent was evaporated to dryness, and the residue was re-dissolved in basic water (pH adjusted to 11 with aq. NH_4_OH) and the solution was centrifuged. The solution was evaporated to dryness yielding 192 mg (50% yield) of the desired benzylamine **2** (Supplementary Figures 6, 7), which was used in the next step without further purification. ^1^H NMR (600 MHz, D_2_O) δ 8.34 (s, ^1^H), 7.99 (d, *J* = 8.0 Hz, ^1^H), 7.51 (d, *J* = 8.0 Hz, ^1^H), 3.91 (s, 2H). MS (ESI) *m/z*: [M–H]^−^ calcd for C_8_H_7_N_2_O_4_^−^ 195.1; found: 195.1

This method is a significant improvement from the one described in the literature (Lee et al., 2004) due to the absence of side products. When 4-(bromomethyl)-3-nitrobenzoic acid reacts with liquid ammonia in ethanol, yielding amine **2**, the alcohol counterpart is formed as a byproduct. This side product is believed to be carried out over the course of the synthetic route, since it is very difficult to separate it from the desired amine, and it could potentially be carried out into the solid phase reactions. Therefore, the method described here *via* an intermediate azide **1** confers a much cleaner method.

##### 4-({[(4-Methylphenyl)diphenylmethyl]amino}methyl)-3-nitrobenzoic Acid (3)

A reported procedure (Lee et al., 2004) was adapted as follows: in a round bottom flask, 4-methyltrityl chloride (2.9 g, 10.2 mmol) was dissolved in a mixture of anh. chloroform (20 ml) and anh. DMF (10 ml) and the mixture was stirred vigorously. The benzylamine **2** (2.0 g, 10.2 mmol) was added to the reaction mixture at room temperature and the reaction mixture was stirred for 30 min. Distilled and anh. triethylamine (2 equiv, 2.8 ml) was added dropwise. The reaction progress was monitored by TLC. After a total of 130 min the reaction mixture was evaporated to dryness. After automated flash chromatography (40 g silica, hexane to ethyl acetate, then to methanol, Supplementary Figure 8), the pure desired protected amine **3** was obtained (1.4 g of 31% yield, Supplementary Figures 9, 10). ^1^H NMR (500 MHz, acetone-*d*_6_) δ 8.49 (s, ^1^H), 8.40–8.34 (m, 2H), 7.56 (d, *J* = 9.2 Hz, 4H), 7.43 (d, *J* = 8.3 Hz, 2H), 7.31 (t, *J* = 7.2 Hz, 4H), 7.21 (t, *J* = 7.3 Hz, 2H), 7.13 (d, *J* = 8.5 Hz, 2H), 3.69 (s, 2H), 2.29 (s, 3H). MS (ESI) *m/z*: [M–H]^−^ calcd for C_28_H_23_N_2_O_4_^−^ 451.2; found: 451.2

##### 3-Amino-4-({[(4-methylphenyl)diphenylmethyl]amino}methyl)benzoic Acid (4)

Following a procedure reported in the literature (Lee et al., 2004): in a Schlenk flask, the protected amine **3** (700 mg, 1.55 mmol), ethyl acetate (18 ml), and platinum dioxide (70 mg, 20 mol%) were combined and, under nitrogen atmosphere, the reaction mixture was degassed by using the freeze-pump-thaw method (3×). The atmosphere in the flask was changed to hydrogen by placing an H_2_-filled balloon, connected to a needle which was inserted directly into the solution. The hydrogenation reaction was left stirring for 24 h at 25°C. The reaction progress was monitored by mass spectrometry. The crude material was then filtered with kieselguhr and the filtrate was evaporated under reduced pressure. The desired product **4** was obtained with a quantitative yield (655 mg, Supplementary Figures 11, 12) and was used in the next step without further purification. Spectral data agree with those previously reported. ^1^H NMR (600 MHz, methanol-*d*_4_) δ 7.48 (d, *J* = 8.2 Hz, 4H), 7.36–7.33 (m, 3H), 7.29–7.25 (m, 5H), 7.20–7.15 (m, 3H), 7.10 (d, *J* = 8.1 Hz, 2H), 3.35 (s, 2H), 2.29 (s, 3H). MS (ESI) m/z: [M–H]^−^ calcd for C_28_H_25_N_2_O_2_^−^ 421.1 found: 421.3.

#### Assembly of 1Aa on Solid Phase

##### Coupling of Template Compound to the Rink Amide Resin

The steps leading up to the macrocyclization were adapted from procedures in the literature (Pattarawarapan et al., 2002; Lee et al., 2004; Zaccaro et al., 2005). The Rink amide resin (0.09 mmol, 0.5 mmol/g, 180 mg) was swollen overnight in dry DCM (1.8 ml) in a plastic fritted syringe. To remove the Fmoc protective group, the resin was treated with a solution of 20% (v/v) piperidine in anh. DMF (1.5 ml) for 1.5 h, and then a fresh portion of the same solution (1.5 ml) was added for another h. The resin was washed with solvent following this sequence (1.5 ml each): DMF, MeOH, DMF, MeOH, DCM (2×), MeOH (2×), and DCM (3×). The beads were then drained and carried over to the next step. A solution of template compound **4** (3 equiv); HBTU (3 equiv, 102 mg); HOBt (3 equiv, 36 mg) and DIEA (5 equiv, 79 μl) in anh. DMF (1.5 ml) was added to the fritted syringe carrying the rink resin, and the syringe was gently shaken for 2 h. The solvents were drained, and the resin (**5**) washed.

##### Incorporation of Fmoc-Lys (Boc)-OH

The reaction mixture with **5** was treated with a solution of Fmoc-Lys-(Boc)-OH (4 equiv, 169 mg), PyBrOP (6 equiv, 252 mg) and distilled and anh. 2, 6-lutidine (15 equiv, 157 μl) in dry DCM (1.5 ml) and it was left shaking gently overnight. Then, the solvents of the original crude material were drained, the beads were washed and the Fmoc group cleaved (2 × 1.5 ml, 1 h each turn). The resin (**6**) was then washed as described above.

##### Incorporation of Fmoc-Ile-OH

Resin **6** was treated with a solution of Fmoc-Ile-OH (3 equiv, 95 mg), DIC (3 equiv, 42 μl); HOBt (3 equiv, 36 mg) and anh. DIEA (5 equiv, 79 μl) in 4:1 (v/v) anh. DCM/DMF (1.5 ml) and the vessel was gently shaken for 4 h. The solvents were drained, the beads were washed and the Fmoc group was cleaved with 20% (v/v) piperidine in anh. DMF (2 × 1.5 ml, 10 min first, then 15 min). The resin (**7**) was then washed as described above.

##### Incorporation of 2-fluoro-5-nitrobenzoyl Chloride

Resin** 7** was treated with a solution of 2-fluoro-5-nitrobenzoyl chloride (3 equiv, 55 mg) and anh. and distilled DIEA (3 equiv, 47 μl) in dry DCM (1.5 ml) for 50 min. The reaction was monitored by checking the color of the solution after using the ninhydrin test. The solvents were drained, and the beads were washed. The Mtt protecting group was removed by treatment with a solution of 1% TFA and 5% TIS in DCM (2 ml portions, eight times, or until the yellow color disappeared completely). Lastly, the resin (**8**) was washed as described above.

##### Macrocyclization to 1Aa-NO_2_

Intermediate **8** was treated with a suspension of K_2_CO_3_ (10 equiv, 124 mg) in anh. DMF (2 ml) at 25°C, with gentle shaking, for 2 d. To monitor the cyclization, a small portion of beads were isolated, washed with water, dried under vacuum for 1 h, and then cleaved from the resin with 1 ml of 18:1:1 TFA/TIS/H_2_O for 2 h. The solvent was then evaporated under reduced pressure. LC-MS analysis was then performed on the residue [column: Waters Xterra analytical column, 4.6 × 150 mm; solvent system: A—1% formic acid in water, B—0.1% formic acid in MeCN; program: gradient of 0–100%B over 15 min; flow rate: 1.0 ml/min; UV detection at 254 and 280 nm; *t*_r_ = 8.1 min, Supplementary Figure 13]. After completion of the cyclization, the resin (**9**) was washed as described above and carried over to the next step. MS (ESI) *m/z*: [M + H]^+^ calcd for C_27_H_36_N_7_O_6_^+^ 554.3, found 554.4.

##### Nitro Group Reduction

**9, 13** was treated with 1.5 ml of 2 M SnCl_2_•2H_2_O in anh. DMF for 22 h with gentle shaking. The resin (**10**) was washed with water (3 × 1.5 ml) before washing as above, then dried under vacuum for 1 h and carried over to the next step.

##### (12*S*,15*S*)-20-Amino-12-(4-aminobutyl)-15-(butan-2-yl)-11,14,17-trioxo-2,10,13,16-tetraazatricyclo[16.4.0.0^4,9^]docosa-1(18),4(9), 5,7,19,21-hexaene-7-carboxamide (1Aa, 11)

A solution of 18:1:1 TFA/TIS/H_2_O was added to the syringe containing **10** and the mixture was gently shaken for 2 h. The solvent was drained, and the resin was rinsed with water (3×) and ethyl ether (3×). The solution was evaporated to dryness. The final product (1Aa, **11**) was purified by semi-preparative reversed-phase HPLC (column: Hamilton PRP-1, 10 × 250 mm, 7 μm; solvent system: A—5% MeCN with 0.1% formic acid, B—10% MeCN with 0.1% formic acid; program: 0%B for 20 min, 100%B for 15 min; flow rate: 3.5 ml/min; UV detection at 280 nm; *t*_r_ = 25.9 min, Supplementary Figure 14) to obtain the title compound (7.4 mg, 16% overall yield, Supplementary Figure 16), with an estimated purity of 90%. The purity was confirmed by LC–MS analysis (column: Waters Xterra analytical column, 4.6 × 150 mm; solvent system: A—2% MeCN with 0.1% formic acid, B—10% MeCN with 0.1% formic acid; program: 0%B for 10 min, 100%B for 15 min; flow rate: 1.0 ml/min; UV detection at 254 and 280 nm; *t*_r_ = 25.9 min, Supplementary Figure 15). ^1^H NMR (400 MHz, D_2_O) δ 7.95 (s, ^1^H), 7.79–7.73 (m, 2H), 7.28 (s, ^1^H), 7.21 (d, *J* = 8.6 Hz, ^1^H), 7.10 (d, *J* = 8.8 Hz, ^1^H), 4.36–4.29 (m, 2H), 4.21–4.14 (m, ^1^H), 4.02–3.94 (m, ^1^H), 3.64–3.56 (m, ^1^H), 3.03 (t, *J* = 7.6 Hz, 2H), 2.05–1.91 (m, 2H), 1.85–1.64 (m, 6H), 1.04 (d, *J* = 6.7 Hz, 3H), 1.03–0.93 (m, 3H). MS (ESI) *m/z*: [M + H]^+^ calcd for C_27_H_38_N_7_O_4_^−^ 524.3; found: 524.3.

#### Synthesis of Boc-1Aa-linker

##### Incorporation of Fmoc-GABA-OH

The synthesis of resin-immobilized 1Aa (**10**) was scaled up using 320 mg (0.16 mmol) of starting resin. **10** was then treated with a mixture of Fmoc-GABA-OH (156.1 mg, 3 equiv), HBTU (182.0 mg, 3 equiv), and DIEA (110 μl, 5 equiv) in anh. DMF (2.5 ml) for 15 h. After washing the resin following the above sequence, the Fmoc group was removed with 1:4 (v/v) piperidine/DMF (2 × 1.5 ml, 1 h each). The resin (**12**) was then washed as above.

##### Reaction With Succinic Anhydride

Resin **12** was treated with succinic anhydride (480.3 mg, 36 equiv) in anh. DMF (2.5 ml) for 21 h. The resin (**13**) was then washed as described above.

##### 3-[(3-{[(12*S*,15*S*)-15-[(2*S*)-Butan-2-yl]-12-(4-{[(*tert*-butoxy)carbonyl]amino}butyl)-7-carbamoyl-11,14,17-trioxo-2,10,13,16-tetraazatricyclo [16.4.0.0^4,9^]docosa-1(22),4(9),5,7,18,20-hexaen-20-yl]carbamoyl}propyl)carbamoyl]propanoicacid (Boc-1Aa-linker, 14)

**13** was treated with 18:1:1 (v/v) TFA/TIS/H_2_O (3 ml) for 3 h. The resin beads were removed by filtration and the yellow filtrate was evaporated under reduced pressure, leaving an oily residue which was treated with cold diethyl ether (5 ml), causing precipitation of a yellow solid. After centrifugation, the precipitate was washed with cold diethyl ether (2 × 5 ml), then dried under vacuum. The crude cleavage product was obtained as a yellow solid (39.5 mg) and used in the following step without further purification. MS (ESI) *m/z* [M–H]^−^: calcd for C_35_H_47_N_8_O_8_^−^ 707.4; found 707.5.

The product was dissolved in 1:2 (v/v) anh. DMF/MeOH (9.0 ml) and to the solution was added di(*tert*-butyl) dicarbonate (74 μl, 2 equiv) and TEA (22 μl, 1.1 equiv) with stirring. After stirring overnight at room temperature, the solution was then evaporated under reduced pressure. The residue was taken up in 2:1 DMF/water (2 ml), then centrifugated. The solution of crude product was purified by semi-preparative reversed-phase HPLC [column: Hamilton PRP-1, 21.2 × 250 mm, 7 μm; solvent system: A—20% MeCN, 50 mM TEAB (pH 7.8), B—75% MeCN, 50 mM TEAB (pH 7.8); program: 0%B for 5 min, gradient of 0–34%B over 30 min, then an isocratic hold at 100%B for 10 min; flow rate: 8 ml/min; UV detection at 260 nm; *t*_r_ = 27.4 min] to afford **14** as a light-yellow powder (26.8 mg, 20% from starting resin, Supplementary Figures 17, 18). ^1^H NMR (400 MHz, DMSO-*d*_6_) δ 9.70 (s, ^1^H), 9.64 (s, ^1^H), 8.33 (s, ^1^H), 8.16 (d, *J* = 8.7 Hz, ^1^H), 7.91 (s, ^1^H), 7.85 (t, *J* = 5.5 Hz, ^1^H), 7.68 (d, *J* = 7.4 Hz, ^1^H), 7.56 (s, 2H), 7.48 (d, *J* = 2.5 Hz, ^1^H), 7.41 (dd, *J* = 8.8, 2.5 Hz, ^1^H), 7.30 (s, ^1^H), 6.76 (d, *J* = 5.8 Hz, ^1^H), 6.69 (d, *J* = 8.9 Hz, ^1^H), 6.00 (br. s, ^1^H), 4.64 (dd, *J* = 14.7, 6.4 Hz, ^1^H), 4.35 (q, *J* = 6.8 Hz, ^1^H), 4.20 (dd, *J* = 15.2, 3.3 Hz, ^1^H), 3.95 (t, *J* = 9.2 Hz, ^1^H), 3.04 (q, *J* = 6.7 Hz, 2H), 2.89 (q, *J* = 6.6 Hz, 2H), 2.39 (t, *J* = 6.9 Hz, 2H), 2.28 (t, *J* = 6.9 Hz, 2H), 2.23 (t, *J* = 7.4 Hz, 2H), 1.85–1.56 (m, 7H), 1.43–1.34 (m, 1^1^H), 1.30–1.22 (m, 2H), 0.93–0.86 (m, 6H). MS (ESI) *m/z*: [M–H]^−^ calcd for C_40_H_55_N_8_O_10_^−^ 807.4; found 807.6.

#### Coupling to Ris-linker

##### Activation and Reaction With Ris-linker

To a solution of **14** (27.8 mg, 34 μmol) in anh. DMF (0.8 ml) was added TEA (4.2 μl, 2 equiv), followed by a freshly prepared 0.05 M solution of TSTU in anh. DMF (300 μl, 1 equiv). After stirring for 2 h at room temperature, the solution of the crude NHS ester intermediate **15**, MS (ESI) *m/z*: [M + Na]^+^ calcd for C_44_H_59_N_9_O_12_Na^+^ 928.4; found 928.6 was used directly in the next step.

The “magic-linker” procedure (Sun et al., 2016) was adapted to that step as follows: Ris-linker (**16**) (2× TEA salt, 14.0 mg, 2.5 equiv) was dissolved in water (1.2 ml) and the pH was set to 7.5 using Na_2_CO_3_. The DMF solution of the crude intermediate (**15**) was added to the aqueous Ris-linker solution dropwise with stirring and the pH of the solution was readjusted to 8.4 with Na_2_CO_3_. Stirring was continued for 3 h, then the mixture was centrifugated. The pellet was then taken up in 0.05 M TEAB (1:4 (v/v) MeCN/water, pH 7.6, 500 μl) and the mixture was centrifuged. The supernatants containing the crude product were filtered through a 0.45 μm syringe filter, then purified on a semi-preparative reversed-phase HPLC [column: Hamilton PRP-1, 10 × 250 mm, 7 μm; solvent system: A—20% MeCN, 50 mM TEAB (pH 7.8), B—75% MeCN, 50 mM TEAB (pH 7.8); program: 0%B for 5 min, gradient of 0–25%B over 20 min, then an isocratic hold at 60%B for 10 min; flow rate: 2.5 ml/min; UV detection at 260 nm; *t*_r_ = 24.8 min, Supplementary Figure 19] to yield the TEA salt (1.5×) of the conjugate (**17**) as a white powder (10.1 mg, 52% over 2 steps, Supplementary Figures 20–22). ^1^H NMR (400 MHz, D_2_O, pD 6.71) δ 8.76 (s, ^1^H), 8.55 (d, *J* = 6.4 Hz, ^1^H), 8.44 (d, *J* = 7.4 Hz, ^1^H), 7.97 (s, ^1^H), 7.85 (t, *J* = 6.9 Hz, ^1^H), 7.73–7.63 (m, 2H), 7.45 (s, ^1^H), 7.28 (d, *J* = 8.6 Hz, ^1^H), 6.94 (d, *J* = 8.9 Hz, ^1^H), 4.36–4.11 (m, 6H), 3.48–3.32 (m, 5H), 3.29–3.23 (m, 2H), 3.08 (t, *J* = 6.4 Hz, 2H), 2.59–2.48 (m, 4H), 2.41 (t, *J* = 6.9 Hz, 2H), 1.99–1.65 (m, 7H), 1.61–1.48 (m, 4H), 1.39 (s, 9H), 1.03 (d, *J* = 6.6 Hz, 3H), 0.97 (t, *J* = 7.3 Hz, 3H). ^31^P NMR (162 MHz, D_2_O) δ 16.25 (d, *J* = 25.9 Hz), 16.02 (d, *J* = 26.3 Hz). MS (ESI) *m/z*: [M–2H]^−^ calcd for C_50_H_71_N_10_O_17_P_2_^2-^ 1145.5; found 1145.3.

##### 1-(3-{3-[(3-{[(12*S*,15*S*)-15-[(2*S*)-Butan-2-yl]-12-(4-aminobutyl)-7-carbamoyl-11,14,17-trioxo-2,10,13,16-tetraazatricyclo[16.4.0.0^4,9^]docosa-1(22),4(9),5,7,18,20-hexaen-20-yl]carbamoyl}propyl)carbamoyl]propanamido}-2-hydroxypropyl)-3-(2-hydroxy-2,2-diphosphonoethyl)pyridin-1-ium (Ris-1Aa, 18)

To a solution of **17** in water (600 μl) was added TFA (300 μl). After 3 h, the solution was evaporated under reduced pressure, then the residue was taken up in 10% MeCN in water (0.05 M TEAB, pH 9.0). The mixture was then centrifugated, and the supernatant was purified by semi-preparative reversed-phase HPLC [column: Hamilton PRP-1, 10 × 250 mm, 7 μm; solvent system: A—10% MeCN, 50 mM TEAB (pH 9.0), B—75% MeCN, 50 mM TEAB (pH 9.0); program: 0%B for 5 min, gradient of 0–25%B over 20 min, then an isocratic hold at 60%B for 10 min; flow rate: 3 ml/min; UV detection at 260 nm; *t*_r_ = 18.0 min, Supplementary Figure 23] to yield the final product (Ris-1Aa, **18**) as an off-white powder (4.8 mg, 57%, Supplementary Figures 24, 25); the amount of total bisphosphonate was quantified by ^31^P NMR using pamidronate as the external standard and H_3_PO_4_ in D_2_O (capillary) as the internal standard. Purity was confirmed by LC–MS [column: Hamilton PRP-C18, 4.6 × 150 mm, 7 μm; solvent system: A—10% MeCN, 50 mM TEAB (pH 9.0), B—50% MeCN, 50 mM TEAB (pH 9.0); program: gradient of 0–100%B over 20 min; flow rate: 0.8 ml/min; UV detection at 266 nm; *t*_r_ = 6.3 min, Supplementary Figure 26]. ^1^H NMR (400 MHz, D_2_O, pD 8.9) δ 8.76 (s, ^1^H), 8.51 (d, *J* = 7.7 Hz, ^1^H), 8.33 (d, *J* = 9.2 Hz, ^1^H), 7.98 (s, ^1^H), 7.80 (t, *J* = 8.0 Hz, ^1^H), 7.72–7.63 (m, 2H), 7.44 (s, ^1^H), 7.25 (d, *J* = 8.6 Hz, ^1^H), 6.92 (d, *J* = 9.2 Hz, ^1^H), 4.36–4.22 (m, 3H), 4.21–4.10 (m, 2H), 3.48–3.39 (m, 2H), 3.34–3.22 (m, 6H), 2.96 (t, *J* = 6.6 Hz, 2H), 2.60–2.47 (m, 4H), 2.44–2.33 (m, 2H), 2.02–1.92 (m, 2H), 1.92–1.79 (m, 3H), 1.77–1.64 (m, 3H), 1.55–1.38 (m, 3H), 1.02 (d, *J* = 6.3 Hz, 3H), 0.96 (t, *J* = 6.8 Hz, 3H).^ 31^P NMR (162 MHz, D_2_O, pD 8.9) δ 16.50 (d, *J* = 20.3 Hz), 16.30 (d, *J* = 20.3 Hz). MS (ESI) *m/z*: [M–2H]^−^ calcd for C_45_H_63_N_10_O_15_P_2_^2−^ 1045.4; found 1045.5.

### *In vitro* Spiral Ganglion Neurite Outgrowth Model

The animal portion of this study was conducted according to the NIH Guide for the Care and Use of Laboratory Animals and approved by the Institutional Animal Care and Use Committee (IACUC) at Mass Eye and Ear.

CBA/CaJ pups at postnatal day (p4) were decapitated, and cochleae were extracted from temporal bones and otic capsules in Hank’s Balanced Salt Solution (HBSS, Life Technologies). The stria vascularis and sensory epithelium were removed, leaving the modiolus containing SGNs, which were then horizontally and vertically dissected into six pieces.

Sterilized round glass coverslips were added to 4-well dish plates (CellStar). Coverslips were then covered with 1:10 dilution of Matrigel (Corning) in culture medium [DMEM/F12 (GIBCO), N2 (ThermoFisher), B27 (Thermofisher), 50 μl/ml Ampicillin (Sigma-Aldrich), 1:300 Fungizone (250 μg/ml, GIBCO), and 1:100 Hepes (1 M, GIBCO)] and placed in the incubator at 37°C for 10 min. Plates were washed with HBSS. The divided modiolus pieces containing SGNs were allowed to attach to glass coverslips overnight in 50 μl of culture medium at 37°C, 5% CO_2_. All drugs were stored as stock solutions in DMSO at 400 μM at −20°C. After tissue attachment was confirmed under the microscope, SGNs were treated with Risedronate, 1Aa, Ris-1Aa, or DMSO medium alone (control) at final concentrations of 400 nM in darkness due to light sensitivity of compounds. DMSO was used for drug dilution at a ratio of 1: 5 - DMSO : water, final DMSO concentration in culture was < 1:1,000. Cultures were kept at 37°C for an additional 48 h after drug applications.

For analysis of phosphorylated TrkC expression, we included treatment with TrkC antibody (Santa Cruz, #sc517245) to block the receptor. SGNs were plated as described above, and additional conditions included pretreatment of SGNs with TrkC antibody at 4 μg/ml for 24 h, followed by 24 h of 1Aa 400 nM treatment, or simultaneous treatment of SGNs with 1Aa at 400 nM and TrkC antibody at 4 μg/ml.

For comparison of neurite outgrowth between 1Aa and DHF, *n* = 4. In cases of outgrowth with Ris-1Aa compared to Ris and 1Aa *n* = 7, and for HA nanoparticle bound outgrowth, *n* = 4. The outgrowth study for phosphorylated TrkC was performed *n* = 3 times. Neurite outgrowth varied in degree based on the compound. In control samples, on average 10–30 neurites were traceable per picture. In treated samples, generally more neurites were found to grow out of the tissue sample, between 20–60 neurons.

Samples were fixed with 4% paraformaldehyde in phosphate-buffered saline (PBS) for 10 min, washed with PBS, then permeabilized and blocked with blocking solution (15% goat serum and 0.3% Triton X-100 in PBS) for 1 h at room temperature. Primary antibody for mouse anti-TUJ1 (1:300, Biolegend, #801201) to detect neurons, or phosphorylated TrkC, pTrkC (1:50, Sigma, #SAB4504648) were diluted in antibody solution (10% goat serum and 0.1% Triton X-100 in PBS) was applied to cultures overnight at 4°C. Samples were washed three times, then incubated with secondary antibody, goat anti-mouse Alexa Fluor 568 (1:500, Invitrogen, #A-11004) diluted in antibody solution for 1 h at room temperature. The tissues were washed three times with PBS. Nuclei were labeled with DAPI (1:1,000, BD Biosciences) diluted in PBS. After washing again as above, coverslips were mounted on glass slides using Fluoromount-G Medium (Invitrogen) and sealed with clear nail polish (Electron Microscopy Sciences). The tissues were then visualized with Leica Sp8 confocal microscopy at 10 ×.

### *In vitro* Hydroxyapatite Outgrowth Assays

Hydroxyapatite (HA) nanopowder (10 mg, Sigma-Aldrich) was suspended in 1 ml of culture medium and filtered through a 40 μm sterile filter to remove agglutinated nanoparticles. Four 1 ml suspensions of HA, each containing ~500 μg of nanopowder, in culture medium were prepared in the dark. 6-Fam-Ris (8 nM, 1:50 relative to other compounds added, BioVinc) was added to each suspension. Risedronate, 1Aa, Ris-1Aa, and DMSO alone at final concentrations of 400 nM were added to each dilution, briefly vortexed, then incubated at 37°C for 1 h.

Particles were centrifuged at 2,000 rpm for 2 min and the supernatant was removed. Residues were washed four times with a fresh culture medium. HA pellets were then resuspended in 40 μl of 1:5 Matrigel in the culture medium. The Matrigel-HA solution was spread onto sterilized round glass coverslips and placed in the incubator at 37°C for 15 min.

CBA/CaJ pups (p4) were euthanized and modiolus containing SGNs were extracted as described above. Tissues were plated onto solidified Matrigel-HA suspension for 48 h. Outgrowth was analyzed using immunocytochemistry and confocal imaging as described above.

### Neurite Tracing Analysis

Neurite tracing for soluble and HA SGN outgrowth assays was performed blinded to treatment identities. z-stacks were imported to ImageJ and the Simple Neurite Tracer plug-in was used to trace neurite paths through the z-stack. Only neurites with visible start and endpoints were traced. Paths were then compiled into a skeleton, and average neurite lengths were compared between treatments.

### *In vitro* Cochlear Synaptopathy Model

CBA/CaJ pups (p4) were euthanized and cochleae were extracted from temporal bones and otic capsules as described above. The stria vascularis was removed leaving the organ of Corti attached to the modiolus. The middle turn was isolated and cut into two pieces using breakable scalpel blades and a blade holder (Fine Science Tools).

Explants were plated on 1:10 Matrigel pretreated 4-well dish plates with sterilized round glass coverslips as described above. Explants attached onto coverslips overnight in culture medium. After confirming attachment under the microscope, explants were treated in darkness with 0.5 mM kainic acid (KA, Abcam) diluted in the culture medium in the incubator at 37°C for 2 h. An untreated explant was kept as a negative control. KA was removed and explants were washed with culture medium. KA treated cultures were treated in darkness with either the 1st group (1Aa, DHF, DHF, and 1Aa, or DMSO alone) or the 2nd group of drugs (Ris, 1Aa, Ris-1Aa, or, DMSO alone). All drugs were diluted to a final concentration of 400 nM. Synapse experiments for comparison of 1Aa and DHF were performed *n* = 4 times, and synapse experiments with Ris, 1Aa, and Ris-1Aa were performed *n* = 10 times.

Samples were fixed and blocked with blocking solution (5% goat serum, 0.3% Triton X-100 in PBS) in preparation for immunocytochemical analysis as described above. Mouse (IgG1) anti-CtBP2 (1:200, BD Biosciences, #612044) for presynaptic ribbons, mouse (IgG2a) anti-PSD95 (1:1,000, Neuromab, #75-028) for postsynaptic neural densities, and rabbit anti-myosin VIIa (1:500, Proteus, #25-6790) for hair cells, diluted in antibody solution (1% goat serum, 0.3% Triton X-100 in PBS) were applied to samples overnight at room temperature. After washing in PBS three times, Alexa Fluor antibodies (1:500 in antibody solution), goat anti-mouse IgG2a 488 (Invitrogen, #A-21131), goat anti-mouse IgG1 568 (Invitrogen, #A-21124), goat anti-rabbit 647 (Invitrogen, #A-21244), were applied for 1 h at room temperature. After another washing step, coverslips were mounted on glass slides as described above. Explants were visualized with Leica SP8 confocal microscope at 63× with an additional 3.17× zoom.

### Synaptic Ribbon Quantification and Colocalization Analysis

Image analysis was performed blinded to treatment identities. z-stacks were imported to Amira imaging software (Visage Imaging, version 6.4). Isosurface functions and connected components were used to create 3D renderings of the images and synaptic colocalizations were analyzed as previously described (Suzuki et al., 2016; Kempfle et al., 2018).

### Statistics

This work was conducted with support from the Biostatistics Program at Harvard Catalyst | The Harvard Clinical and Translational Science Center (National Center for Advancing Translational Sciences, National Institutes of Health Award UL 1TR002541) and financial contributions from Harvard University and its affiliated academic healthcare centers. The content is solely the responsibility of the authors and does not necessarily represent the official views of Harvard Catalyst, Harvard University and its affiliated academic healthcare centers, or the National Institutes of Health. Statistical analysis was performed using Student’s *t*-test, one-way and two-way ANOVA with Tukey’s multiple comparisons test in PRISM software (Version 9.0). A *p*-value less than 0.05 was considered statistically significant.

## Results

We synthesized a TrkC agonist, 1Aa, to assess its neurotrophic activity. In a second step, we utilized synthetic chemistry to create a novel small molecule, Ris-1Aa, that linked 1Aa to risedronate (Ris), a bisphosphonate with high bone mineral affinity. We studied both molecules for their ability to promote neurite outgrowth and ribbon synapse regeneration *in vitro*.

### Synthesis of the Small Molecule TrkC Agonist, 1Aa

**Figure 1:**
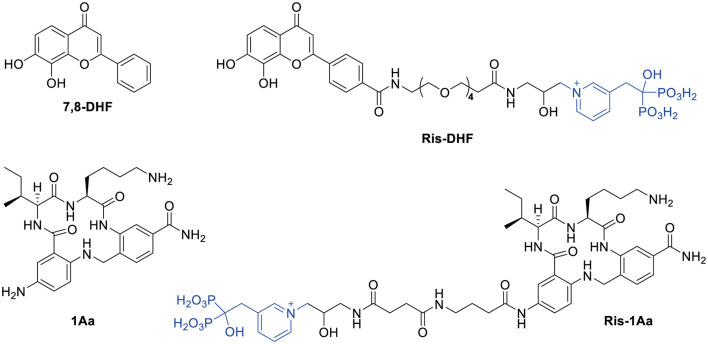
Structures of 7,8-DHF, 1Aa, and their conjugates with risedronate (Ris).

For our investigations of the biochemical activity of 1Aa, we first prepared the compound by solid-phase synthesis (Figure 1; Supplementary Figure 1). Starting from 4-(bromomethyl)-3-nitrobenzoic acid (**1**), we prepared the corresponding 4-(4-methyltritylaminomethyl-3-aminobenzoic acid (**4**) in four steps by a modification of the literature procedure (Lee et al., 2004). Assembly of 1Aa was initiated by coupling **4** to Rink amide resin, giving the resin-attached intermediate (**5**, Supplementary Figure 1, Step e). This was reacted with ε-Boc-α-Fmoc protected lysine, giving **6** which after removal of the Fmoc with piperidine, was condensed with Fmoc-protected isoleucine, providing after deprotection at the Ile α-amino group the dipeptide derivative (**7**). After reaction with 2-fluoro-5-nitrobenzoyl chloride and macrocyclization, the nitro group on the resulting intermediate (**9**) was reduced to an amino group by SnCl_2_ in DMF, yielding resin-bound 1Aa (**10**). Reactions involving resin-bound compounds were monitored by cleavage of a portion of the sample with TFA, then analysis by LC-MS. Treatment of **10** with 1:1:18 H_2_O/TIS/TFA followed by removal of the resin beads and evaporation of the filtrate afforded crude 1Aa (**11**) which was isolated as its formate salt after semi-preparative reverse-phase HPLC and characterized by ^1^H NMR and LC-MS.

### Design and Synthesis of the Ris-1Aa Conjugate

**Figure 2:**
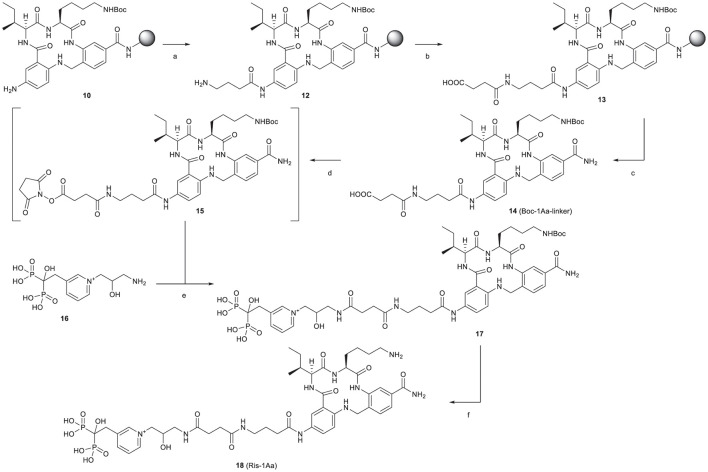
Synthesis of Ris-1Aa. Reagents and conditions: **(A)** (1) Fmoc-GABA-OH, HBTU, DIEA, DMF, 15 h (2) 20% piperidine in DMF; **(B)** succinic anhydride, DMF, 25 h; **(C)** (1) 1:1:18 H_2_O/TIS/TFA, 3 h; (2) Boc_2_O, TEA, 1:2 DMF/MeOH, 22 h, 20% over 14 steps; **(D)** TSTU, TEA, DMF, r.t., 2 h; **(E)** NaHCO_3_, 2:3 DMF/H_2_O, pH 8.3, 19 h, 52% over 2 steps; **(F)** 1:2 TFA/H_2_O, 3 h, 57%.

The synthetic route is outlined in Figure 2. Boc-protected 1Aa immobilized on the resin (**10)** was first decorated with a Fmoc-protected γ-aminobutanoic acid (GABA) linker, which was then deprotected with piperidine to give **12** and further modified by reaction with succinic anhydride to give **13**. After cleavage from the resin, Boc protection was reinstalled on the lysine ε-amine in the 1Aa moiety and the terminal carboxyl function of the resulting intermediate **14** was activated as an unisolated *N*-hydroxy succinimidyl ester **15**, which was reacted *in situ* with risedronate equipped at the pyridyl nitrogen with a 3-(2-hydroxy)-1-aminopropyl (**16**) to form **17**. After deprotection of **17** with aqueous TFA, the final product (**18**, Ris-1Aa) was purified by reversed-phase HPLC and characterized by ^1^H NMR ^31^P NMR and LC-MS.

### 1Aa Stimulates Neurite Outgrowth *In vitro*

**Figure 3:**
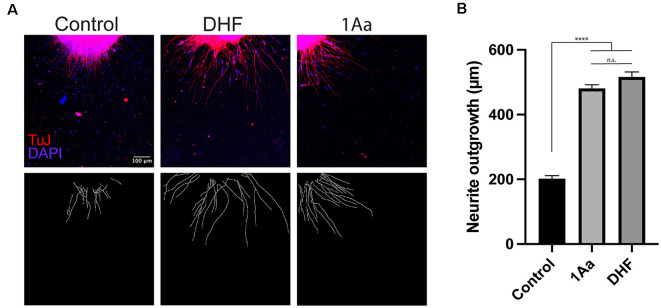
1Aa promotes neurite outgrowth *in vitro*. **(A)** SGN neurite outgrowth *in vitro*. Neurites were stained with neuronal marker TuJ (red), and nuclei were labeled with DAPI (blue). Scale bar represents 100 μm. Images are representative of four independent experiments. **(B)** Quantification of neurite outgrowth in μm compared to DMSO control and DHF (n.s., not significant; ^****^ represents *p* ≤ 0.0001).

In order to compare the effect of 1Aa on neurite outgrowth to our previously published results on DHF, we treated postnatal SGNs in culture with 400 nM of 1Aa, DHF, a combination of equal amounts of DHF and 1Aa, or control with DMSO for 48 h. Immunohistochemistry for neural marker TuJ was performed and TuJ positive neurites were quantified. The average neurite outgrowth ranged between 100 nm and 600 nm. Compared to control, outgrowth significantly increased by 2- to 2.5-fold for DHF and 1Aa and DHF + 1Aa treated samples (Figure 3). There was no significant increase in neurite outgrowth upon treatment with both DHF and 1Aa. In summary, these results suggest that 1Aa can promote spiral ganglion neurite outgrowth *in vitro* to a level comparable to DHF.

### Ris-1Aa Promotes Neurite Growth *In vitro*

**Figure 4:**
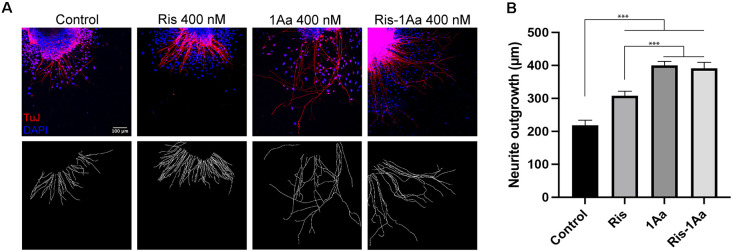
Ris-1Aa supports neurite outgrowth *in vitro* in a manner comparable to 1Aa. **(A)** SGN neurite outgrowth *in vitro*. Neurites were stained with neuronal marker TuJ (red), and nuclei were labeled with DAPI (blue). Scale bar represents 100 μm. Images are representative of seven independent experiments. **(B)** Quantification of neurite outgrowth compared to DMSO control (^***^ represents *p* ≤ 0.001).

We next analyzed the ability of Ris-1Aa to promote neurite outgrowth. We treated postnatal SGNs in culture with 400 nM of Ris, 1Aa, and Ris-1Aa, or control with DMSO for 48 h, and quantified the lengths of axons staining positive for TuJ (Figure 4A). Average neurite outgrowth ranged between 100 nm to 600 nm. Relative outgrowth for Ris-1Aa was comparable to our prior experiments with 1Aa (Figure 4B). This finding suggests that the neurotrophic activity of 1Aa *in vitro* is not abrogated by conjugation with Ris.

### Ris-1Aa Promotes Neurite Outgrowth After Pre-binding to Hydroxyapatite

**Figure 5:**
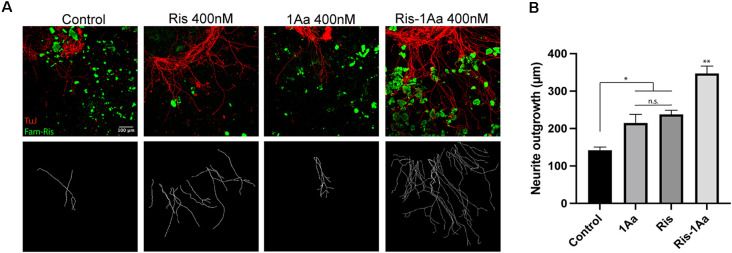
Ris-1Aa retains neurotrophic activity following binding to the bone matrix *in vitro*. **(A)** SGN neurite outgrowth after preincubation of drugs with hydroxyapatite nanoparticles (HA). Neurites were stained with neuronal marker TuJ (red), HA was visualized with Fam-Ris (green), and nuclei were labeled with DAPI (blue). Scale bar represents 100 μm. Images are representative of four independent experiments. **(B)** Quantification of neurite outgrowth compared to DMSO control (* represents *p* ≤ 0.05, ** represents *p* ≤ 0.01; n.s., not significant).

We have previously established an assay to assess the activity of novel hybrid compounds containing bisphosphonates conjugated with neurotrophic small molecules, following binding to hydroxyapatite bone matrix (Kempfle et al., 2018). Ris-1Aa or control molecules were pre-bound to hydroxyapatite nanoparticles and assessed for their ability to promote SGN neurite outgrowth (Figure 5A). Ris-1Aa demonstrated the strongest outgrowth compared to control, Ris, or 1Aa (Figure 5B). A small positive effect on outgrowth was seen by Ris alone, which is consistent with our prior data. The modest outgrowth demonstrated in response to 1Aa may be from trapping within the hydroxyapatite particles after rinsing.

### 1Aa Acts at Least in Part Through TrkC *In vitro*

**Figure 6:**
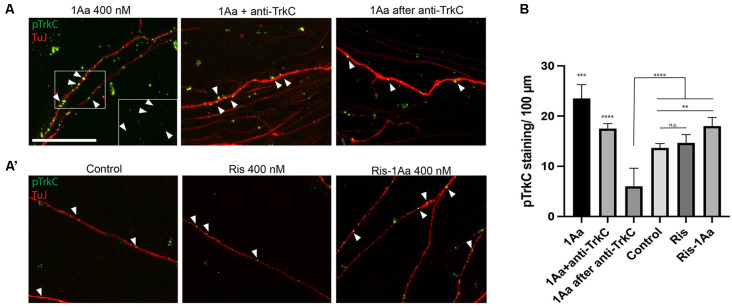
1Aa and Ris-1Aa activate TrkC *in vitro*. **(A)** and **(A′)** Expression of phosphorylated TrkC (pTrkC) on SGN neurites *in vitro*. Neurites were stained with neuronal marker TuJ (red), and phosphorylated TrkC (green), and nuclei were labeled with DAPI (blue). Scale bar represents 10 μm. The outgrowth of 1Aa was compared to pre-treatment with a blocking anti-TrkC antibody (1Aa after anti-TrkC) or simultaneous treatment with 1Aa and anti-TrkC (1Aa + TrkC). **(A′)** pTrkC staining for control, Ris or Ris-1Aa of the same experiment. Images are one representative experiment of three independently performed experiments. Inlay and arrowheads in **(A)** and **(B)** mark examples of pTrkC staining. **(B)** Quantification of pTrkC per 100 μm. 1Aa and 1Aa+anti-TrkC were each significant compared to all other conditions (^**^represents *p* ≤ 0.01, ^***^ represents *p* ≤ 0.001, ^****^ represents *p* ≤ 0.0001; n.s., not significant).

To provide mechanistic insight into whether 1Aa acts *via* TrkC in primary auditory neurons, we treated SGNs in our organotypic culture model with either 1Aa alone, in combination with a blocking antibody specific to the extracellular portion of TrkC, or following pretreatment with the blocking antibody. To assess for activation of TrkC receptors in SGNs, we stained for phosphorylated TrkC after treatment and quantified the number of phosphorylated receptors found on each neurite (Figures 6A,A′,B). We compared the number of phosphorylated receptors to untreated SGNs, or SGNs treated with Ris or Ris-1Aa (Figures 6A′,B). We demonstrated that, in the presence of 1Aa, the number of phosphorylated receptors increased significantly compared to control or samples treated with the blocking anti-TrkC antibody. Pretreatment with the anti-TrkC antibody resulted in the lowest number of phosphorylated receptors on the neurites across all treatment conditions. Ris-1Aa also demonstrated activity through TrkC, although the amount of phosphorylated TrkC appeared to be lower than after treatment with 1Aa. Interestingly, the number of phosphorylated TrkC receptors on neurites treated with Ris alone was not significantly different compared to control, suggesting that Ris does not act through TrkC (Figure 6B).

### 1Aa Stimulates Regeneration of Cochlear Ribbon Synapses *In vitro*

**Figure 7:**
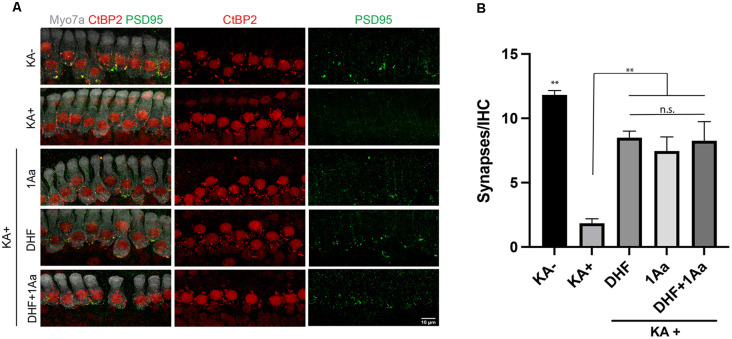
1Aa promotes synapse regeneration *in vitro*. **(A)** Immunohistochemistry of untreated control (KA-), control treated with KA (KA+), explant treated with KA+DHF (DHF), explant treated with KA+1Aa (1Aa), and explant treated with KA and 1Aa+DHF (1Aa+DHF). Hair cells were labeled with Myo7A (white), presynaptic ribbon synapse was labeled with CtBP2 (red), and postsynaptic ribbon synapse was stained with PSD95 (green). Images are representative of four independent experiments. Scale bar represents 10 μm. **(B)** Quantification of number of synapses per inner hair cell (^**^ represents *p* ≤ 0.01, n.s., not significant).

To assess synaptic regeneration after 1Aa treatment, we isolated organ of Corti (OC) explants with intact ribbon synapses and SGNs for *in vitro* culture (Figure 7A). After treatment with kainic acid (KA), which induces excitotoxic damage to ribbon synapses, OC explants were treated with 400 nM of 1Aa, DHF, 1Aa + DHF, or DMSO (control). Quantification of intact synapses per IHC (Figure 7B), as measured by the proximity of pre- and post-synaptic markers, revealed an average synapse count of 11–12 synapses for the untreated control OC explants (KA-). KA-treated OC explants without any regenerative treatment (KA+) averaged around two synapses. Treatment with DHF, 1Aa or 1Aa + DHF demonstrated a significant and statistically similar increase in synaptic counts after KA treatment, with an average number of 8–10 synapses per IHC. These data suggest that the small molecule TrkC agonist 1Aa has the ability to regenerate ribbon synapses following KA damage *in vitro*, comparable to the previously described effect of DHF. Furthermore, there does not appear to be an additive effect of both TrkB and TrkC stimulation for increasing synaptic counts under these experimental conditions.

### Ris-1Aa Promotes Synaptic Regeneration *In vitro*

**Figure 8:**
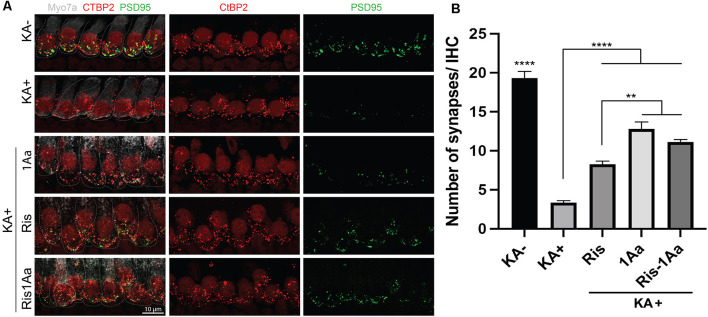
Ris-1Aa promotes synapse regeneration *in vitro*. **(A)** Immunohistochemistry of untreated control (KA-), control treated with KA (KA+), explant treated with KA+ 1Aa, explant treated with KA+ Ris, and explant treated with KA and Ris-1Aa. Hair cells were labeled with Myo7A (white), presynaptic ribbon synapse was labeled with CtBP2 (red), and postsynaptic ribbon synapse was stained with PSD95 (green). Images are representative of 10 independent experiments. Scale bar represents 10 μm. **(B)** Quantification of synapses per inner hair cell for various treatment conditions (^****^p ≤ 0.0001, denotes that counted synapses in both KA- and KA+ were statistically significant relative to all other samples; ^**^p ≤ 0.01).

Finally, we evaluated the ability of the bisphosphonate conjugate Ris-1Aa to promote synaptic regeneration. As above, we treated OC explants *in vitro* with KA to destroy synapses to inner hair cells (Figure 8A). OC explants were then treated with 400 nM of 1Aa, Ris, Ris-1Aa, or DMSO (control). Pre- and postsynaptic portions were quantified and demonstrated a significant increase in synaptic regeneration after treatment with Ris-1Aa (Figure 8B). This was comparable to numbers of synapses obtained after treatment with 1Aa. These data suggest that Ris-1Aa maintains the ability of 1Aa to increase synaptic counts following KA treatment.

## Discussion

We report that neurotrophin-3 small molecule analogue 1Aa promotes SGN outgrowth and regeneration of inner ear ribbon synapses *in vitro*. These effects were similar to those of DHF, a small molecule analogue of BDNF. Bone-binding hybrid molecule Ris-1Aa retained most of native 1Aa’s neurotrophic ability *in vitro*, both when freely available in culture and when pre-bound to hydroxyapatite. Our results suggest that 1Aa and Ris-1Aa may be attractive candidates to promote the regeneration of cochlear ribbon synapses *in vivo*.

These data are the first to establish the activity of an NT-3 small molecule analogue, 1Aa, upon SGNs. Previous work identified 1Aa in a screen of small molecule peptidomimetics capable of activating TrkA and/or TrkC. This report defined 1Aa as a TrkC agonist as it bound TrkC, induced TrkC phosphorylation, and promoted differentiation of a cell line expressing TrkC. 1Aa also potentiated the activity of NT-3 (Zaccaro et al., 2005). Here, we extend these findings to demonstrate that 1Aa and Ris-1Aa have neurotrophic activity on mature SGNs *in vitro* to induce neurite elongation and regeneration of synapses between IHCs and SGNs. Moreover, treatment of SGNs with 1Aa and Ris-1Aa induced TrkC phosphorylation, which is the first step in TrkC signaling following ligand binding. Therefore, it is reasonable to conclude that the effects on SGNs we observe are, at least in part, due to activity through TrkC. Our present data do not, however, rule out that 1Aa and Ris-1Aa also activate TrkB. Critically, stimulation *via* TrkC has been shown in various systems to be superior to TrkB stimulation with respect to cochlear synaptic regeneration (Wang and Green, 2011; Wan et al., 2014). In this regard, DHF, a small molecule TrkB agonist, can partially restore cochlear ribbon synapses *in vivo* following noise damage, although this effect requires a high concentration of DHF directly injected into perilymph *via* a labyrinthotomy (Fernandez et al., 2021). 1Aa, by acting at least in part through TrkC, may exhibit a more powerful regenerative effect *in vivo*.

Two of the primary challenges of inner ear drug delivery are the complex anatomy of the labyrinth and the labyrinth’s existence as a closed, bone-encased system that restricts access *via* the round (RW) and oval (OW) windows and the blood-labyrinth barrier (Swan et al., 2008). Prior approaches have focused on systemic or local delivery to the inner ear. While systemic administration may be relatively straightforward, it has the potential for greater side effects and insufficient drug levels delivered to the inner ear. In cases where hearing loss is not a concern, a local direct approach *via* a cochleostomy affords the potential to maximize drug delivery in a highly controlled manner. However, opening the cochlea presents a risk in patients with residual hearing, as the disturbance of inner ear fluid homeostasis can lead to significant hearing and balance loss. Therefore, local intratympanic delivery to the RW and OW may be a preferred procedure in patients with at least some preserved hearing. In this regard, intratympanic injection, including steroids for sudden hearing loss and gentamicin for Meniere’s disease, is commonly performed in human patients. Previous studies have suggested, however, that the percentage of drug that ultimately enters the inner ear *via* these approaches may be low, ranging between 0.1–2%, and bioavailability depends on multiple factors such as size, hydrophobicity, and distribution processes (Salt and Plontke, 2009).

To address these limitations, we have developed a drug delivery platform for the inner ear that relies on bisphosphonate conjugation to maximize co-localization of neurotrophic activity with SGNs. Bisphosphonates have a high affinity for hydroxyapatite, and drug-bisphosphonate conjugates exploit this chemical property to specifically target bone (Sedghizadeh et al., 2017; Farrell et al., 2018; McKenna et al., 2020). We have previously shown that a fluorescently-labeled bisphosphonate can cross the RWM and avidly label the osseous spiral lamina, which is in close proximity to SGNs (Kang et al., 2015). The use of bisphosphonate conjugates to target the cochlea may also have the advantage of prolonged binding to the bony labyrinth and enable long-term stimulation of SGNs (Kempfle et al., 2018). As we have now described novel small conjugated molecules with neurotrophic activity *in vitro*, we anticipate that other small molecules with desired activities within the cochlea could potentially be delivered *via* this platform. Additional work, perhaps utilizing modified hybrid molecules with the ability to release the neurotrophin analogues from the bisphosphonate, may be needed to optimize neurotrophic delivery and activity.

In this regard, our data describe a general synthetic chemistry approach to conjugate a variety of small molecules with a range of bisphosphonates. This approach allows for fine-tuning of delivery. These novel applications derive from our previously reported procedures for the conjugation of fluorescent dyes to bisphosphonates (Kashemirov et al., 2008; Sun et al., 2016). The design for Ris-1Aa includes a long-chain linker between the 1Aa moiety and risedronate, and the attachment point we used to add the linker and conjugate to Ris ensured that the 1Aa moiety retained its TrkC agonism its neurotrophic activity. We successfully incorporated the linker during solid-phase synthesis of 1Aa, then adapted our established “magic linker” method procedure to conjugate 1Aa to Ris.

Interestingly, in our experimental system Ris itself has the intrinsic ability to drive the regeneration of ribbon synapses *in vitro*. We were the first to show that some bisphosphonates have intrinsic activity and can support ribbon synapse regeneration *in vitro* (Kempfle et al., 2018), a finding that we recapitulate with the present results. This result was unexpected, although recent work replicates these findings and further suggests that some bisphosphonates can promote ribbon synapse regeneration *in vivo* following noise damage (Seist et al., 2020). The mechanisms by which bisphosphonates exert this activity remain unclear. Indeed, the broad spectrum of bisphosphonate activity outside the skeletal system is an active area of investigation (Panagiotakou et al., 2020). Our present data and our previous work (Kempfle et al., 2018), however, strongly suggest that this effect occurs independently of TrkB or TrkC signaling as Ris does not appear to induce either TrkB or TrkC phosphorylation *in vitro*.

We present here the first demonstration that an NT-3 small molecule analogue and its bisphosphonate-linked derivative have neurotrophic activity on SGNs *in vitro*. Our findings may hold important implications for the regeneration of cochlear ribbon synapses, as well as for intralabyrinthine drug delivery more generally.

## Data Availability Statement

The raw data supporting the conclusions of this article will be made available by the authors, without undue reservation.

## Ethics Statement

The animal study was reviewed and approved by the Institutional Animal Care and Use Committee (IACUC) at Mass Eye and Ear, Boston, MA.

## Author Contributions

JK: biological study design, acquisition of biological data, data analysis, and manuscript preparation. CA: compound synthesis and manuscript preparation. AZ: acquisition of biological data, data analysis, and manuscript preparation. MD: compound synthesis and manuscript preparation. RK: data analysis and preparation of manuscript. RL: data analysis. BK: synthesis design, and manuscript preparation. AE: manuscript preparation. CM: project concept, synthesis design, data analysis, and manuscript preparation. DJ: project concept, biological study design, data analysis, and manuscript preparation. All authors contributed to the article and approved the submitted version.

## Conflict of Interest

DJ receives compensation and stock options as a consultant for Akouos. Akouos is a participant in the Department of Defense Grant listed under Funding, but was not involved in this study. The remaining authors declare that the research was conducted in the absence of any commercial or financial relationships that could be construed as a potential conflict of interest.
