# Supplementary material for: A Novel Small Molecule Neurotrophin-3 Analogue Promotes Inner Ear Neurite Outgrowth and Synaptogenesis In vitro
Source: Front Cell Neurosci. 2021 Jul 15;15:666706. doi: 10.3389/fncel.2021.666706 (PMC8319950; doi:10.3389/fncel.2021.666706)
Supplement: Supplementary file 1 [file Data_Sheet_1.docx]

Supplementary Material

# Supplementary Data

## Supplementary Figures

**Supplementary Figure 1*.*** Synthesis of 1Aa. Reagents and conditions: a) NaN_3_, DMF, 12 h, quant.; b) PPh_3_, THF/H_2_O, 12 h, 50%; c) MttCl, CHCl_3_/DMF, 130 min, 31%; d) H_2_ (balloon), PtO_2_, EtOAc, 24 h, quant.; e) HBTU, DIEA, DMF, 5 h; f) 1) Fmoc-Lys(Boc)-OH, PyBrOP, 2,6-lutidine, DCM, 14 h 2) 20% piperidine in DMF; g) 1) Fmoc-Ile-OH, DIC, HOBt, DIEA, 4:1 DCM/DMF, 19 h 2) 20% piperidine in DMF, 2 h; h) 1) 2-fluoro-5-nitrobenzoyl chloride, DIEA, DCM, 50 min; 2) 1:5:94 TFA/TIS/DCM; i) K_2_CO_3_, DMF, 2 d; j) SnCl_2_·2H_2_O, DMF, 22 h; k) 5:5:90 H_2_O/TIS/TFA, 2 h, 16% over 10 steps.


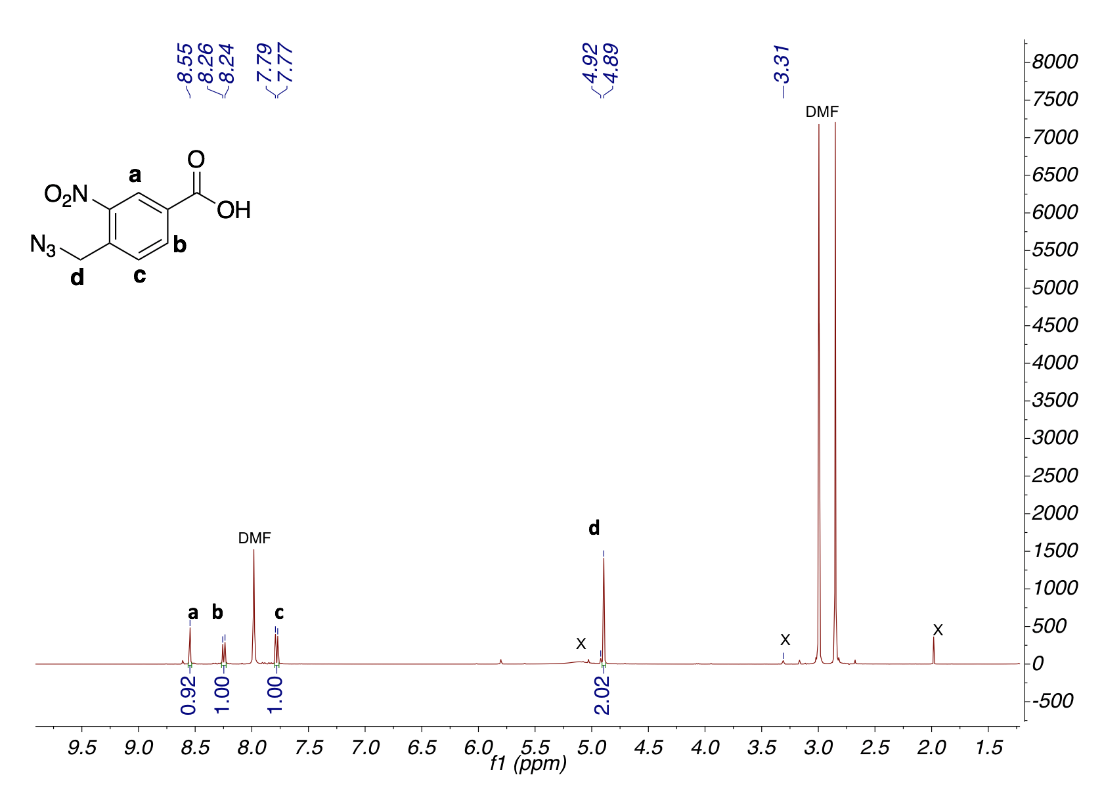


**Supplementary Figure 2.** ^1^H NMR spectrum of **1** (400 MHz, methanol-*d*_4_).


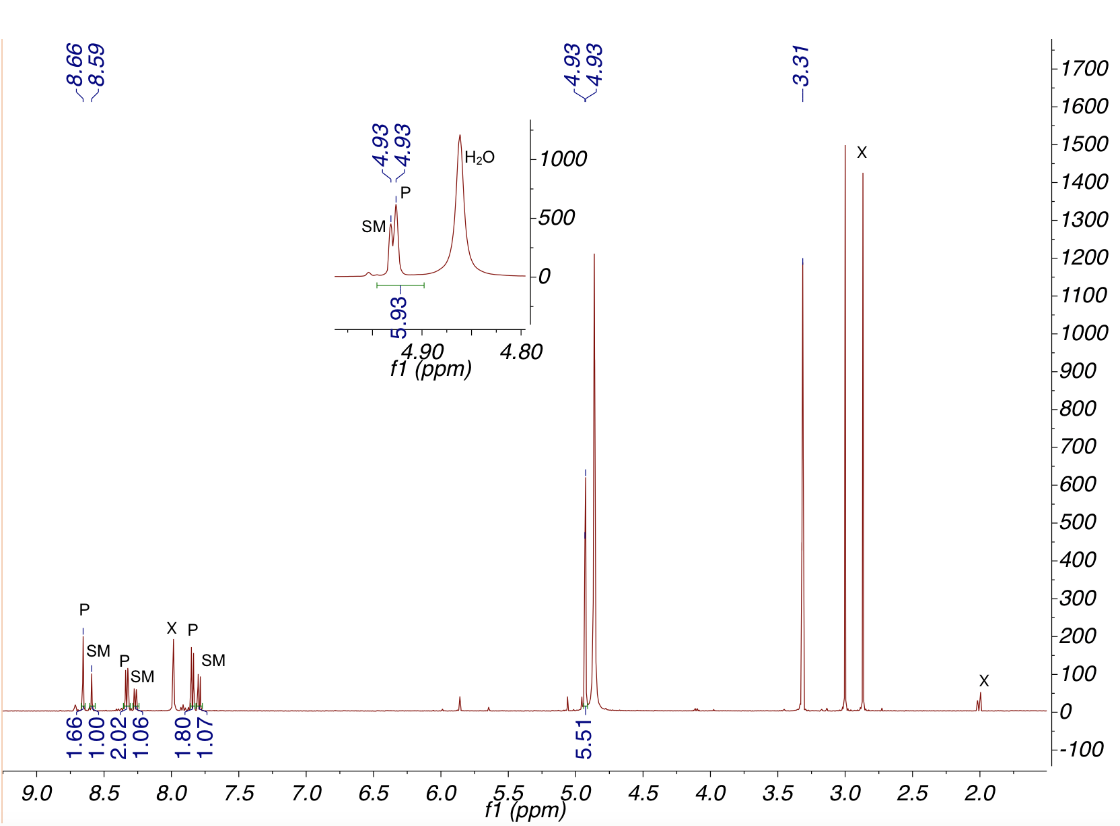


**Supplementary Figure 3.** ^1^H NMR (500 MHz, methanol-*d*_4_) studies of the different chemical shift between the desired product **1** (**P**) and the starting material (**SM**) 4‐(bromomethyl)‐3‐nitrobenzoic acid.

**Supplementary Figure 4.** ^13^C{^1^H} NMR spectrum of **1** (101 MHz, methanol-*d*_4_).


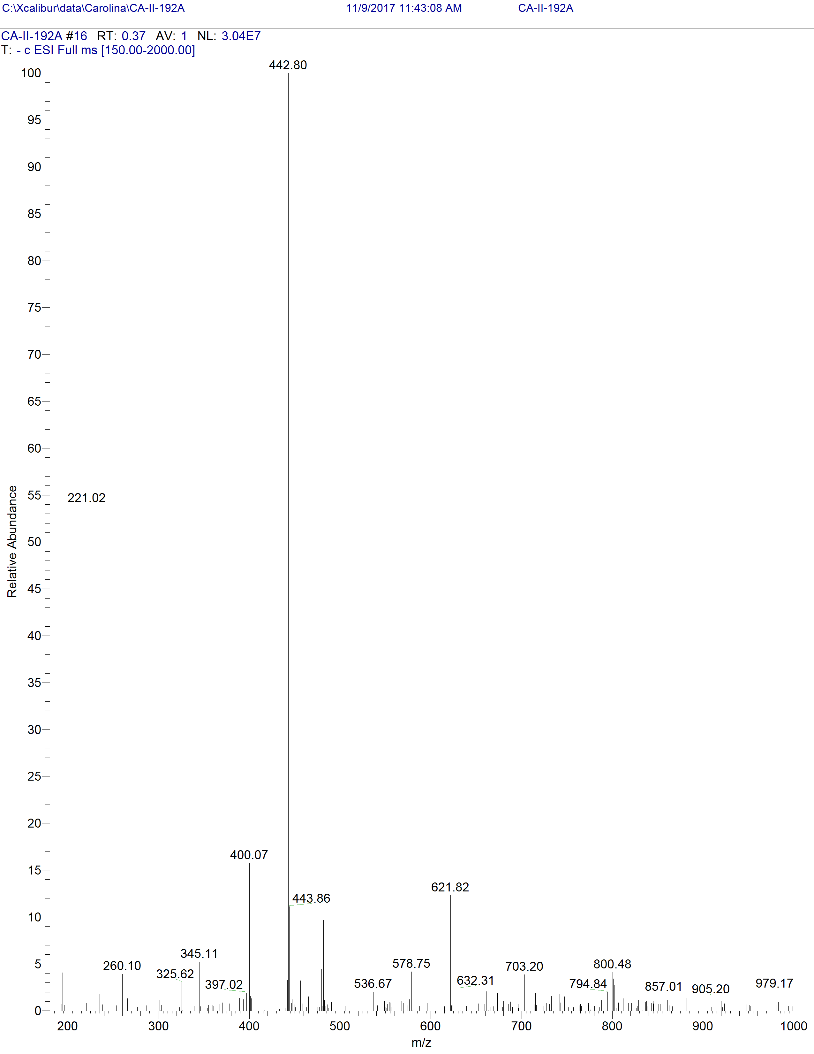


**Supplementary Figure 5.** Mass spectrum (ESI, negative mode) for **1.**

**Supplementary Figure 6.** ^1^H NMR spectrum of **2** (600 MHz, D_2_O, pD 10.7)

**Supplementary Figure 7*.*** Mass spectrum (ESI, negative mode) for compound **2**.

**Supplementary Figure 8*.*** ISCO chromatography of compound **3**. Conditions: solvent A- Hexane, solvent B- ethyl acetate, solvent B4- methanol, flow 40 mL/min, silica column 40g, wavelength 1 (red)- 254nm, wavelength 2 (purple)- 280nm, ELSD detector (green). Product was isolated from fractions 48–60.

**Supplementary Figure 9.** ^1^H NMR spectrum of **3** (500 MHz, acetone-*d*_6_).

**Supplementary Figure 10.** Mass spectrum (ESI, negative mode) for **3**.

**Supplementary Figure 11.** ^1^H NMR spectrum of 4 (600 MHz, methanol-*d*_4_)

**Supplementary Figure 12.** Mass spectrum (ESI, negative mode) for compound **4**.

**Supplementary Figure 13.** LC–MS trace of 1Aa-NO_2_, from cleaved **9**. Mass spectrum (positive mode) for the peak at 8.13 min.

**Supplementary Figure 14.** Semi-preparative RP-HPLC of **11** (*t*_r_ = 25.9 min). Conditions: 3.5 mL/min, 254 nm, gradient mode, A/ 0.1% Formic acid 5% Acetonitrile in H_2_O and B/ 0.1% Formic acid 10% Acetonitrile in H_2_O: 0 to 20 min (A), 20 to 40 min (B).

**Supplementary Figure 15.** LC–MS trace of **11**. Mass spectrum (positive mode) for the peak at 14.11 min.

**Supplementary Figure 16.** ^1^H NMR spectrum of **11** (400 MHz, D_2_O).

**Supplementary Figure 17.** ^1^H NMR spectrum of **14** (400 MHz, DMSO-*d*_6_).

**Supplementary Figure 18.** Mass spectrum (ESI, negative mode) for **14**.

**Supplementary Figure 19.** Semi-preparative RP-HPLC of **17** (*t*_r_ = 24.8 min). Conditions: column: Hamilton PRP-1, 10 × 250 mm, 7 µm; solvent system: A—10% MeCN, 0.1 M TEAB (pH 8), B—75% MeCN, 0.1 M TEAB (pH 8); program: isocratic hold at 0% for 5 min, then a gradient from 0–30%B over 20 min, then 60%B over 10 min; flow rate: 2.5 mL/min.

**Supplementary Figure 20.** ^1^H NMR spectrum of **17** (400 MHz, D_2_O).

######

**Supplementary Figure 21.** ^31^P{^1^H} NMR spectrum of **17** (162 MHz, D_2_O).

**Supplementary Figure 22.** Mass spectrum (ESI, negative mode) for **17**.

**Supplementary Figure 23.** Semi-preparative RP- HPLC of **18** (*t*_r_ = 18.0 min). Conditions: column: Hamilton PRP-1, 10 × 250 mm, 7 µm; solvent system: A—10% MeCN, 0.1 M TEAB (pH 8), B—75% MeCN, 0.1 M TEAB (pH 8); program: isocratic hold at 0% for 5 min, then a gradient from 0–25%B over 20 min, then 60%B over 10 min; flow rate: 3 mL/min.

**Supplementary Figure 24.** ^1^H NMR of **18** (400 MHz, D_2_O). M – methanol; A– acetonitrile.

**Supplementary Figure 25.** ^31^P{^1^H} NMR of **18** (162 MHz, D_2_O).

**Supplementary Figure 26.** LC–MS trace of **18**. Mass spectrum (negative mode) shown for the peak at 6.4 min.
